# Genomic Evidence for Formate Metabolism by *Chloroflexi* as the Key to Unlocking Deep Carbon in Lost City Microbial Ecosystems

**DOI:** 10.1128/AEM.02583-19

**Published:** 2020-04-01

**Authors:** Julia M. McGonigle, Susan Q. Lang, William J. Brazelton

**Affiliations:** a School of Biological Sciences, University of Utah, Salt Lake City, Utah, USA; b School of Earth, Ocean, and Environment, University of South Carolina, Columbia, South Carolina, USA; University of California, Davis

**Keywords:** *Chloroflexi*, hydrothermal vents, metagenomics, methanogens, serpentinization

## Abstract

The Lost City hydrothermal field on the Mid-Atlantic Ridge supports dense microbial life on the lofty calcium carbonate chimney structures. The vent field is fueled by chemical reactions between the ultramafic rock under the chimneys and ambient seawater. These serpentinization reactions provide reducing power (as hydrogen gas) and organic compounds that can serve as microbial food; the most abundant of these are methane and formate. Previous studies have characterized the interior of the chimneys as a single-species biofilm inhabited by the Lost City *Methanosarcinales*, but they also indicated that this methanogen is unable to metabolize formate. The new metagenomic results presented here indicate that carbon cycling in these Lost City chimney biofilms could depend on the metabolism of formate by *Chloroflexi* populations. Additionally, we present evidence for metabolically diverse, formate-utilizing *Sulfurovum* populations and new genomic and phylogenetic insights into the unique Lost City *Methanosarcinales*.

**IMPORTANCE** Primitive forms of life may have originated around hydrothermal vents at the bottom of the ancient ocean. The Lost City hydrothermal vent field, fueled by just rock and water, provides an analog for not only primitive ecosystems but also potential extraterrestrial rock-powered ecosystems. The microscopic life covering the towering chimney structures at the Lost City has been previously documented, yet little is known about the carbon cycling in this ecosystem. These results provide a better understanding of how carbon from the deep subsurface can fuel rich microbial ecosystems on the seafloor.

## Abstract (precis)

Primitive forms of life may have originated around hydrothermal vents at the bottom of the ancient ocean. The Lost City hydrothermal vent field, fueled by just rock and water, provides an analog for not only primitive ecosystems but also potential extraterrestrial rock-powered ecosystems. The microscopic life covering the towering chimney structures at the Lost City has been previously documented, yet little is known about the carbon cycling in this ecosystem. These results provide a better understanding of how carbon from the deep subsurface can fuel rich microbial ecosystems on the seafloor.

## INTRODUCTION

The towering carbonate chimneys of the Lost City hydrothermal field protrude from the Atlantis Massif, a dome of ultramafic rock uplifted from the mantle. These chimneys differ from other deep-sea hydrothermal systems because they are driven primarily by rock-water reactions, known as serpentinization, rather than magmatic activity. The serpentinization reactions create high-pH fluids that mix with the surrounding cold seawater to form the calcium carbonate structures. Serpentinite-hosted systems are of astrobiological interest because they provide a source of energy for life that does not require sunlight or vigorous magmatic activity (1). These systems are thought to be present on icy worlds, such as Jupiter’s moon Europa and Saturn’s moon Enceladus (2, 3).

The dense microbial biofilms of Lost City chimneys are fueled by the carbon and energy released by serpentinization of the underlying ultramafic rock (4–8). The serpentinization reactions provide high concentrations of hydrogen gas, methane, and other simple organic compounds that serve as food and energy sources for microbes. The more extreme interiors of chimneys are anoxic and continually bathed in the warm serpentinizing fluids. The temperatures of venting fluids can reach >95°C, and the pH of the fluids can be as high as 11 (9). Previous studies have shown that these interiors are dominated by a single archaeal phylotype, the Lost City *Methanosarcinales* (5). In contrast, the chimney exteriors host a more complex microbial community, including organisms involved in the oxidation of sulfur and methane (e.g., *Methylomonas*, *Thiomicrospira*). These organisms likely thrive in the mixing zones, where they can take advantage of the cooling effect of the seawater and more efficient electron acceptors (e.g., oxygen) but still access the products of serpentinization supplied by venting fluids.

In general, little is known about the metabolic capabilities of Lost City organisms. Our previous research has shown that much of the microbial biomass at Lost City is derived from carbon that originated deep in the Earth’s subsurface (4, 10). In most ecosystems, inorganic carbon (CO_2_) serves as the starting carbon source for primary production. However, the Lost City fluids contain extremely low concentrations of dissolved inorganic carbon (DIC) due to its reduction to hydrocarbons and its rapid precipitation as calcium carbonate at a pH above ∼9 (11–13). The organic acid formate has been proposed to be an alternative primary carbon source; it is present in high concentrations in Lost City fluids (36 to 158 μM) and is expected to form abiotically in serpentinizing fluids (4, 14, 15). In support of this, our previous experiments have shown that the isotopic compositions of carbon (^13^C and ^14^C) in bacterial and archaeal lipids resemble those of formate from the vent fluids (4).

Formate is unable to enter carbon fixation pathways directly and needs to be converted to CO_2_ for autotrophic metabolism (16–19). The enzyme formate dehydrogenase catalyzes the reversible oxidation of formate to CO_2_. Obtaining formate from the environment requires active transport; therefore, any Lost City formate-utilizing species would carry genes encoding a formate transporter and formate dehydrogenase. This study identifies two formate-utilizing populations of the Lost City chimneys based on metagenomic evidence, including the presence of formate transporters and formate dehydrogenases. These formate-utilizing organisms may enable mantle-derived carbon to become available to the other microbial inhabitants of Lost City chimneys that are unable to use formate, such as the *Methanosarcinales*.

## RESULTS AND DISCUSSION

### Metagenomic assembly and binning.

We performed shotgun paired-end sequencing of environmental DNA extracted from a sample of chimney material collected at Marker 5 within the Lost City hydrothermal field. The metagenome consisted of 145,937,844 read pairs (after quality filtering), which were assembled into 730,351 contigs with an *N*_50_ of 2,518 bp and a maximum length of 250,900 bp. The assembled contigs represent 62.47% of all read pairs in the metagenome. Each contig of >1,000 bp was assigned a taxonomy by the PhyloPythiaS+ program (20), and the overall taxonomic composition of these contigs is shown in Fig. S1 in the supplemental material. These results are consistent with those of previous studies that have described the chimney biofilm communities as being dominated by *Gammaproteobacteria*, including *Thiotrichales* and *Methylococcales* (6, 7, 21).

Contigs were binned into metagenome-assembled genomes (MAGs), only seven of which initially contained <10% contamination and >18% completeness (Table S1) after automated binning. None of these seven MAGs contained strong evidence for formate utilization. Therefore, we manually explored the other MAGs with evidence of formate metabolism.

Two types of formate transporters have been characterized. In formate-utilizing methanogens, the gene *fdhC* is thought to be necessary for the transport of formate into the cell (22). A different formate/nitrite transporter (*focA*) from the same FNT protein family has been described in Escherichia coli (23). E. coli requires *focA* for removal of the formate produced during mixed-acid fermentations, but the protein is known to be bidirectional and can therefore bring formate or nitrite into the cell (24). In order to identify potential formate-using populations in our metagenomes, we examined all bins containing *fdhC* or *focA*. We identified genes for five formate transporters in the metagenome, three of which were found on contigs with evidence of nitrite metabolism, but no other genes involved in formate metabolism. The genes for the other two formate transporters were located on contigs with adjacent genes involved in formate metabolism. Therefore, we manually refined these two MAGs, as well as a third representing the Lost City *Methanosarcinales* phylotype (8, 12).

### *Sulfurovum*.

The *Sulfurovum* MAG was refined to be 95.9% complete and to have 2.19% contamination by examining the hierarchical clustering of contigs, as visualized in the anvi’o platform, and by inspecting the taxonomic assignment (by the PhyloPythiaS+ method) of each contig. The fragments mapped to this MAG comprised 0.41% of the total assembly coverage (Table S2). Of the three MAGs discussed here, the *Sulfurovum* MAG contains the lowest number of protein-encoding genes (2,036), but 90% of these were annotated with a functional prediction. This MAG also has the highest number of complete KEGG modules (25) (Data Set S1).

The *Sulfurovum* MAG includes formate dehydrogenase and formate transporter genes, in addition to genes for a complete KEGG pathway for selenocompound metabolism (ko00450), responsible for synthesizing l-selenocysteinyl-tRNA. The selenocysteine residue is a key feature of the catalytic subunit in formate dehydrogenase (FdhA) and is thought to be directly involved in proton transfer from formate (26).

**FIG 1:**
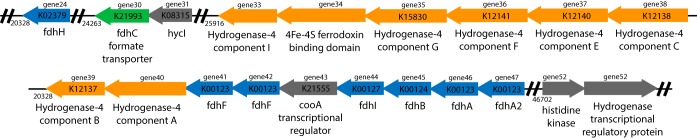
The formate transporter (*fdhC*) contig in the Lost City *Sulfurovum* MAG. Relevant genes are reported here in the order that they are found on the contig. Gene order is indicated above the arrows that indicate the forward or reverse direction of transcription. Double lines indicate where genes are omitted from this visual representation of the contig. Orange genes are involved in the formate hydrogenlyase (FHL) complex; blue genes (*fdhH*, *fdhF*, *fdhI*, *fdhB*, *fdhA*, *fdhA2*) indicate formate dehydrogenase genes that may interact with the FHL complex; the green gene (*fdhC*) is involved in formate transport. All other genes, including regulatory genes, are reported in gray. KEGG identification numbers are indicated on arrows where appropriate. *hycI*, hydrogenase 3 maturation protease; *cooA*, CRP/FNR family CO-sensing transcription factor.

The contig containing the formate transporter gene (*fdhC*) contains multiple formate hydrogenlyase (FHL; hydrogenase-4 [Hyd-4] form) genes starting 1,653 bp away (Fig. 1). Interestingly, the formate hydrogenlyase on this contig is homologous to the Hyd-4 form, which is unaffected by alkaline pH (27). In E. coli, the bidirectional FHL complex links formate oxidation to proton reduction and is operational during mixed-acid fermentations (28). During these fermentations in E. coli, formate is formed by the pyruvate formate-lyase enzyme and transported outside of the cell (29). It is unlikely that the FHL complex is involved in mixed-acid fermentation by *Sulfurovum* because the MAG did not contain genes for a pyruvate formate-lyase. Therefore, FdhC and the FHL complex most likely bring in formate from the environment and carry out the membrane-bound conversion of formate to CO_2_, which can then enter a carbon fixation pathway.

**FIG 2:**
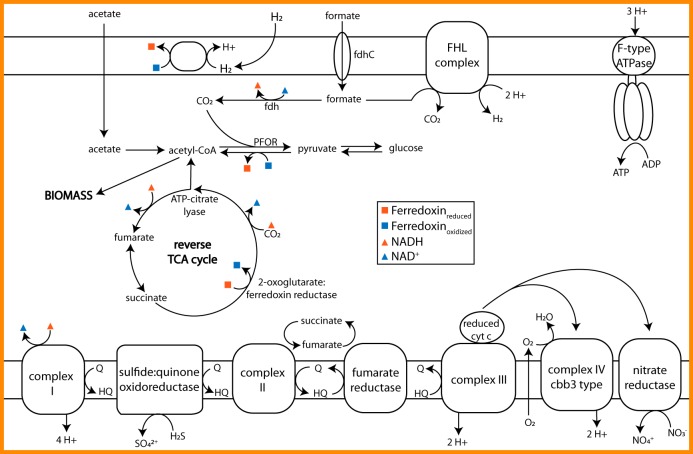
Overview of the Lost City *Sulfurovum* central carbon metabolism pathways and electron transport chain. Abbreviations: fdh, formate dehydrogenase; fdhC, formate transporter; FHL, formate hydrogenlyase complex; HQ, hydroquinone; Q, quinone; cty c, cytochrome *c*; PFOR, pyruvate:ferredoxin oxidoreductase.

Key genes for carbon fixation via the reductive tricarboxylic acid (TCA) cycle were found in the *Sulfurovum* MAG: genes for pyruvate synthase (PFOR; heterodimer type), ATP citrate lyase, 2-oxoglutarate reductase, and fumarate reductase (Fig. 2). In addition, genes for gluconeogenesis are present, suggesting that fixed carbon can be stored as glucose. It is unclear if *Sulfurovum* can run the TCA cycle in the forward direction for heterotrophic growth. Succinate dehydrogenase genes are present in the MAG, but there was no evidence for citrate synthase. Desulfobacter hydrogenophilus is known to use ATP citrate lyase (instead of citrate synthase) in both the forward and the reverse directions, and this may be possible for this *Sulfurovum* population as well (30). Alternatively, instead of using a bidirectional TCA cycle to break down glucose reserves, *Sulfurovum* may ferment it into lactate; indeed, the lactate dehydrogenase gene is present in this MAG.

The *Sulfurovum* MAG contains genes for a complex electron transport chain, suggesting a metabolically diverse lifestyle (Fig. 2). Complex I (NADH dehydrogenase) likely serves as a versatile entry point for many catabolic reactions. We also found a sulfide:quinone oxidoreductase (SQOR), indicating that *Sulfurovum* might use sulfide as an electron donor. Evidence for an electron transport chain in this MAG includes the presence of genes for three terminal electron acceptors: fumarate reductase, cytochrome *c* oxidase (complex IV), and nitrate reductase.

This MAG also contains genes for a number of cofactor ABC transporters, including those for tungstate (required for formate dehydrogenase activity), molybdate, iron, thiamine, and zinc (26). We also found evidence of genes for transporters for macronutrients, such as l-amino acids, branched-chain amino acids, phospholipids, and phosphate.

In addition to these nutrient-acquiring transporters, the MAG contains the gene for a transporter responsible for excreting capsular polysaccharides. After intracellular construction, these molecules are exported to form a capsule around the cell which is involved in both biofilm formation and environmental stress protection (31). We also found a lipopolysaccharide export system, indicating that the *Sulfurovum* population at Lost City builds an outer membrane like other characterized *Sulfurovum* species (32).

Sulfurovum lithotrophicum, the type species for the genus, was first isolated from hydrothermal sediments off the coast of Okinawa, Japan (33). The genome for this species closely resembles our Lost City *Sulfurovum* MAG. It contains genes for a reverse TCA cycle and a sulfide:quinone oxidoreductase (SQOR) and can use O_2_ or NO_3_^−^ as an electron acceptor (34). However, unlike our *Sulfurovum* MAG, this and most characterized *Sulfurovum* species are also able to oxidize sulfur (S^0^) or thiosulfate through the sulfur oxidation (SOX) system (35–37). Both Sulfurovum aggregans and Sulfurovum lithotrophicum have been shown to use hydrogen, but not formate, as an electron donor (36, 38).

The lack of genes for a SOX system and the presence of formate-metabolizing genes in the Lost City *Sulfurovum* MAG are novel for the genus, although it is possible that SOX genes were missed during binning. The genomic capability to scavenge amino acids, form biofilms, and retain genetic flexibility for multiple electron acceptors supports the possibility that this organism has a mixotrophic lifestyle capable of adapting to a fluctuating environment with varying ratios of seawater and hydrothermal fluids. These results suggest that the *Sulfurovum* population is adapted to a transition zone between the interior and the exterior of the chimneys.

### *Chloroflexi*.

After manual refining, the *Chloroflexi* MAG is estimated to be 70% complete with 4.73% contamination. The mapped fragments made up 0.62% of the metagenomic assembly (Table S2). Of the three MAGs discussed here, this MAG contains the highest number of protein-encoding genes (3,936), but only 84% of these were annotated with functional predictions. This bin also has the highest number of incomplete KEGG modules (39) (Data Set 1).

**FIG 3:**
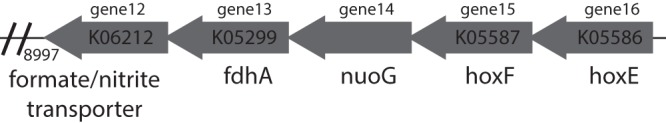
The formate transporter (*focA*) contig found in the Lost City *Chloroflexi* MAG. Note the adjacent gene encoding the alpha subunit of formate dehydrogenase (*fdhA*). Double lines indicate where genes are omitted from this visual representation of the contig. KEGG identification numbers are indicated on arrows where appropriate. *nuoG*, NADH-quinone oxidoreductase subunit G; *hoxF* and *hoxE*, bidirectional (NiFe) hydrogenase diaphorase subunit.

The *Chloroflexi* MAG contains a *focA* formate transporter adjacent to a formate dehydrogenase alpha subunit (*fdhA*) gene and three genes encoding the catalytic subunit of NAD(H) dehydrogenase (*nuoG*, *hoxF*, *hoxE*) (Fig. 3). The beta subunit (*fdhB*) was located together with *fdhA* on a different contig. As with the *Sulfurovum* MAG, the *Chloroflexi* MAG contains genes for a complete KEGG pathway for selenocompound metabolism, responsible for synthesizing l-selenocysteinyl-tRNA.

The *Chloroflexi* MAG contains genes for transporters for tungstate (required for formate dehydrogenase activity), iron, and thiamine. As with *Sulfurovum*, the *Chloroflexi* populations might scavenge macronutrients, as the MAG contains genes for the following transporters: l-amino acids, branched-chain amino acids, phospholipids, and phosphate. Because the MAG is estimated to be only 70% complete, it is likely that it contains genes for additional transporters not identified in this study.

**FIG 4:**
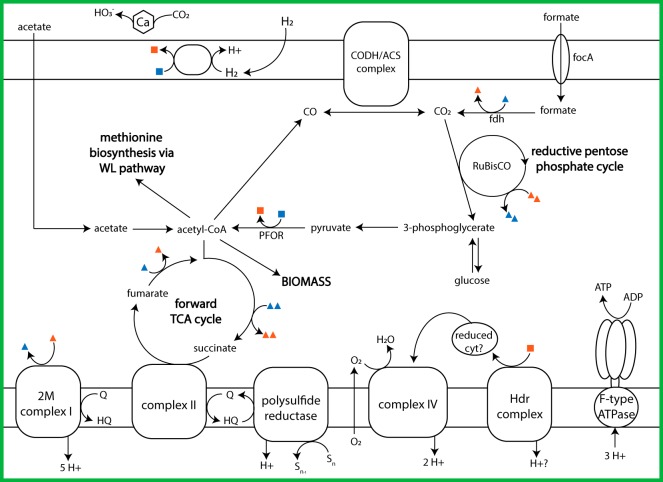
Overview of the Lost City *Chloroflexi* central carbon metabolism pathways and electron transport chain. The definitions of symbols are the same as those provided in the key to Fig. 2. Abbreviations: RuBisCO, ribulose-1,5-bisphosphate carboxylase/oxygenase; Hdr, heterodisulfide reductase; fdh, formate dehydrogenase; CODH/ACS, carbon monoxide dehydrogenase/acetyl-CoA synthase; focA, formate/nitrite transporter; WL pathway, Wood-Ljungdahl pathway; Ca, carbonic anhydrase; cyt, cytochrome; PFOR, pyruvate:ferredoxin oxidoreductase; HQ, hydroquinone; Q, quinone.

In addition to formate-utilizing genes, the *Chloroflexi* MAG contains genes for a nearly complete reductive pentose phosphate cycle (Fig. 4). The MAG also includes genes for a carboxysome-specific carbonic anhydrase, suggesting that this organism uses a carboxysome to concentrate CO_2_ around ribulose-1,5-bisphosphate carboxylase/oxygenase (RuBisCO) (40). Carboxysomes are used by many organisms when the concentration of CO_2_ outside the cell is lower than the *K_m_* of RuBisCO (39). If this *Chloroflexi* couples the carboxysome shell with the conversion of formate to CO_2_, it could be an effective adaptation to the lack of CO_2_ in the chimney environment.

The one enzyme missing from the reductive pentose phosphate cycle in this MAG is glyceraldehyde-3-phosphate dehydrogenase (GAPDH), which is also involved in glycolysis. Interestingly, thermophilic organisms use a distinct form of GAPDH to cope with the heat instability of glyceraldehyde-3-phosphate (30, 41), so it is possible that the *Chloroflexi* MAG contains the gene for an as yet unidentified variant of GAPDH. Another adaptation of thermophilic growth is in the structure of fructose-bisphosphate aldolase, an enzyme involved in gluconeogenesis (41). The gene for this enzyme is also included in the *Chloroflexi* MAG and is most closely related to that of the thermophile Caldilinea aerophila.

The *Chloroflexi* MAG also contains the genes for an incomplete Wood-Ljungdahl pathway. Typically, the presence of genes for this pathway in a bacterial genome indicates that it is involved in carbon fixation during acetogenesis, but the lack of genes for two key enzymes in this MAG casts doubt on that scenario. One of these missing enzymes (methylene-tetrahydrofolate reductase [MTHFR]) is essential for acetogenesis. The second missing enzyme is acetate kinase, which is involved in the last step of acetate formation. While the absence of genes in an incomplete MAG must be interpreted with caution, a similar partial Wood-Ljungdahl pathway has been described in the dehalogenating *Chloroflexi* Dehalococcoides mccartyi (42). Although D. mccartyi is missing MTHFR, the species is capable of *de novo* methionine biosynthesis through the partial Wood-Ljungdahl pathway via the cleavage of acetyl coenzyme A (CoA) synthetase (ACS). As the Lost City *Chloroflexi* MAG contains no genes for other pathways for methionine biosynthesis, this organism, like D. mccartyi, may use the incomplete Wood-Ljungdahl pathway for methionine biosynthesis rather than for carbon fixation.

The *Chloroflexi* MAG has genes for both glycolysis/gluconeogenesis pathways and a forward TCA cycle. This suggests that the organism could store carbon fixed through the reductive pentose phosphate pathway as glucose reserves and grow heterotrophically when carbon is limited in the environment. Additional evidence for a flexible mixotrophic lifestyle for this MAG includes genes for three carbohydrate transporters (multiple sugar, ribose, and d-xylose transport systems), suggesting that this *Chloroflexi* population is capable of metabolizing additional complex carbon sources.

Genomic evidence points to a modified structure of the NADH:quinone oxidoreductase (complex I) in this *Chloroflexi* population. An additional NuoM subunit is responsible for translocating an extra proton per reaction cycle in these modified complexes (43). The operon arrangement for these 2M complexes is unique to cultured *Chloroflexi* species (43). In particular, these species encode an additional *nuoM*_2_, located between the *nuoL* and *nuoM*_1_ genes (as in *nuoLM*_1_*M*_2_*N*), and *nuoBCDI* are separated from the operon with a fused *nuoCD*. These genomic features are consistent with two contigs in our *Chloroflexi* MAG. The *nuoM* sequence, which forms a clade with the modified *nuoM*_2_ gene of Anaerolinea thermophila (Fig. S2), is surrounded by three *nuo* genes: *nuoN*; the *nuoM* sequence, which forms a clade with the *nuoM*_1_ gene of Anaerolinea thermophila; and *nuoL* (as in *nuoLM*_1_*M*_2_*N*). The *nuoBCDI* genes are found on a separate contig in the MAG. The increased proton-pumping ability for the modified 2M complex I has been proposed to be beneficial for energy conservation in alkaliphilic environments or slow-growing organisms (43).

The other membrane-bound complexes encoded by the *Chloroflexi* MAG are also consistent with an anaerobic, mixotrophic lifestyle (Fig. 4). Additional energy conservation appears to be mediated by a modified NADH:quinone oxidoreductase (2M complex I), succinate dehydrogenase (complex II), polysulfide reductase, cytochrome *c* oxidase (complex IV), heterodisulfide reductase, and an F-type ATPase typical of bacteria. Electrons could be donated by formate or carbohydrates, but the terminal electron acceptor is unclear.

The MAG has the gene for cytochrome *c* oxidase (complex IV), which would indicate oxygen as the terminal electron acceptor, but the lack of the gene for cytochrome oxidoreductase, such as a cytochrome *bc* or *b*_6_*f* complex (complex III), is perplexing. The genome of anaerobic Sulfurimonas gotlandica strain GD1 contains the gene encoding cytochrome *c* oxidase, but the enzyme’s suspected function is to occasionally remove inhibitory oxygen rather than to serve as a terminal electron acceptor (44). Furthermore, the *Chloroflexi* genus *Anaerolineae* is described to be obligately anaerobic, yet many species contain genes for aerobic respiration in their genomes (45). Due to the presence of genes encoding numerous oxygen-sensitive enzymes (aldehyde ferredoxin oxidoreductase, the carbon monoxide dehydrogenase [CODH]/ACS complex, anaerobic forms of glycerol-3-phosphate dehydrogenase, and sulfatase maturase) in the *Chloroflexi* MAG, it is likely that the cytochrome *c* oxidase’s role is to remove oxygen rather than to serve in the last step of an aerobic respiratory chain. Alternatively, complex III genes could be missing simply due to the incomplete nature of the MAG.

If complex III is missing, heterodisulfide reductase (Hdr) may be involved in electron transfer from hydrogen (via ferredoxin reduced by hydrogenases) to cytochromes, as proposed for other species (46, 47). This complex could work in tandem with cytochrome *c* oxidase to remove intracellular oxygen. If oxygen is not the terminal electron acceptor, then the presence of polysulfide reductase (Psr) indicates that polysulfide compounds are the most likely terminal electron acceptors. The *Chloroflexi* polysulfide reductase sequence has 40% identity with the Psr sequence from Thermus thermophilus, which has been shown to use polysulfide as its terminal electron acceptor (48).

Considering the presence of multiple genes encoding oxygen-sensitive enzymes and similarities to the genomes of anaerobic organisms, we propose that the *Chloroflexi* population is an anaerobic population adapted to the interiors of Lost City chimneys, where it would have access to abundant formate. By building biomass from formate, *Chloroflexi* populations would convert mantle-derived carbon into organic matter that could subsequently be utilized by other members of the community.

### *Methanosarcinales*.

The manually refined *Methanosarcinales* MAG was estimated to be 84.87% complete with 5.26% contamination. The mapped fragments made up 4.41% of the entire assembly (Table S2). The MAG contained 2,324 protein-encoding genes, only 77% of which could be assigned a predicted function. The *Methanosarcinales* MAG has 33 complete and 28 incomplete KEGG modules (Data Set S1).

**FIG 5:**
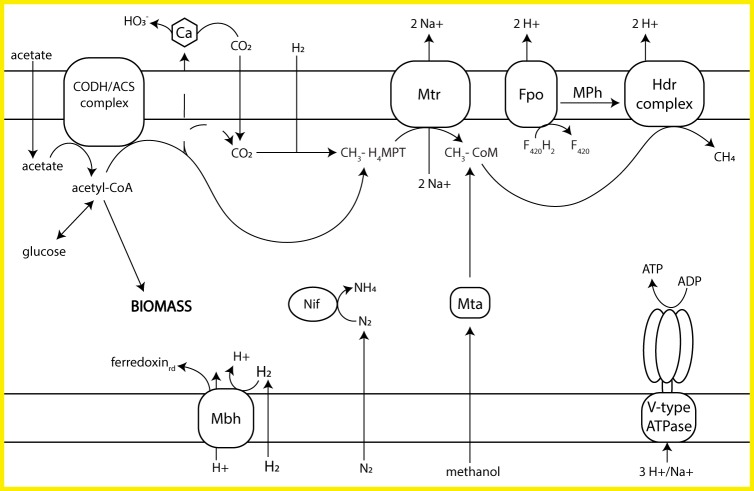
Overview of the Lost City *Methanosarcinales* (LCMS) methanogenic pathway and membrane-bound complexes involved in energy conservation. Abbreviations: CODH/ACS, carbon monoxide dehydrogenase/acetyl-CoA synthase; Mbh, membrane-bound hydrogenase; Nif, nitrogenase; Mtr, methyl-H_4_MPT, coenzyme M methyltransferase; Mta, methanol:5-hydroxybenzimidazolylcobamide methyltransferase; Fpo, F_420_H_2_ dehydrogenase; MPh, methanophenazine; Hdr, heterodisulfide reductase; ferredoxin_rd_, reduced ferredoxin.

We identified this MAG as the previously described Lost City *Methanosarcinales* phylotype due to the taxonomic assignment of *Methanosarcinales* for all contigs and the presence of nitrogenase reductase (*nifH*, nitrogen fixation) and methyl coenzyme M reductase (*mcrA*, methanogenesis) gene sequences that matched those of previously sequenced genes (8, 12) (Tables S3 and S4). In agreement with our previous analysis with a smaller data set, we found no evidence that the *Methanosarcinales* population is able to utilize formate as a carbon source (4). Although novel, previously undiscovered formate metabolism genes may exist, no archaeal formate dehydrogenase gene sequences (*fdhA*, *fdhB*) or *fdhC* sequences affiliated with methanogens were detected in the metagenome. The MAG does contain a genomic inventory that would allow the *Methanosarcinales* to utilize CO_2_, acetate, and methanol for methanogenesis (Fig. 5). The genes for transporters for tungstate and molybdate were also identified; these metals are cofactors required for many of the enzymes in the methanogenic pathway.

The *Methanosarcinales* MAG provides new information on the phylogenetic status of this species. Previous studies classified it within the order *Methanosarcinales* (5), but it has never been maintained as a cultivated isolate, despite many attempts, and a more specific phylogenetic classification has never been attempted. Its closest relatives have been previously reported to include members of the *Methanosarcinaceae* and *Methanosaetaceae* families (5, 7). These two families include the only species known to be capable of methane production from acetate (49). Each group of methanogens has distinct mechanisms for acetate activation and energy conservation. Acetate activation in *Methanosarcina* proceeds via two enzymes: acetate kinase and phosphotransacetylase. *Methanosaeta* species, in contrast, use acetyl-CoA synthetase (ACS) for acetate activation. The Lost City *Methanosarcinales* MAG includes genes for both ACS and acetate kinase (but not the gene for phosphotransacetylase, perhaps due to the incompleteness of the MAG), suggesting that it may be able to use both systems or a hybrid system.

The energy conservation strategy of Lost City *Methanosarcinales* appears to be more similar to that of *Methanosaeta* than to that of *Methanosarcina*. As in *Methanosaeta*, the MAG contains no genes for Ech hydrogenase, the Rnf complex, or the methanophenazine-reducing hydrogenase (Vho). Instead, the only gene for an energy-conserving complex external to the methanogenesis pathway present in the *Methanosarcinales* MAG is that for F_420_H_2_ dehydrogenase, which is also employed by *Methanosaeta* (49).

Phylogenetic analyses of methanogenesis genes from this MAG suggest that it is distinct from both *Methanosaeta* and *Methanosarcina*, perhaps forming a novel family within the order *Methanosarcinales* (Fig. S3). In two of the gene trees, those for formylmethanofuran-tetrahydromethanopterin formyltransferase and F_420_-dependent N^5^,N^10^-methylene- tetrahydromethanopterin reductase (*ftr* and *mer*), the Lost City gene was monophyletic with methylotrophic methanogens, such as Methanococcoides burtonii. A third phylogeny, F_420_-dependent N^5^,N^10^-methylene-tetrahydromethanopterin dehydrogenase (*mtd*), grouped the Lost City gene with hydrogenotrophic and formate-utilizing *Methanosarcinales*, such as Methanothermococcus okinawensis. In a fourth phylogeny, N^5^,N^10^-methenyl-tetrahydromethanopterin cyclohydrolase (*mch*), the Lost City gene was distinct from all other known species.

No genomes of *Methanosaeta* contain genes for membrane-bound hydrogenases that would allow H_2_ to serve as the electron donor (50–52). However, the Lost City MAG contains five genes annotated as ferredoxin-dependent membrane-bound hydrogenase (Mbh). This complex is known to translocate protons with the formation or cleavage of hydrogen gas, similar to the Ech hydrogenase found in many hydrogenotrophic methanogens (53). Therefore, the Lost City *Methanosarcinales* may be able to use this enzyme for hydrogenotrophic methanogenesis. For hydrogenotrophic growth, the Lost City *Methanosarcinales* would have to obtain CO_2_ from the chimney’s carbonate minerals (for which there is no known mechanism) or from another member of the community, such as the *Sulfurovum* or *Chloroflexi* populations described above.

The *Methanosarcinales* MAG contains the *nifH*, *nifD*, and *nifK* genes, encoding the nitrogenase complex involved in nitrogen fixation. Previous work found low δ^15^N values of Lost City chimneys, indicative of biological nitrogen fixation (25). The concentrations of biologically available nitrogen are relatively low (<6 μM) in Lost City fluids, but the concentrations of N_2_ resemble those in seawater (25). Therefore, the densely populated biofilm communities of Lost City chimneys must be reliant on nitrogen fixation, probably carried out at least in part by *Methanosarcinales* populations.

### Conclusion.

The biofilms growing on Lost City chimneys are unique ecosystems where microbes must face the challenges of multiple extremes, including pHs of >10 and temperatures of up to at least 95°C. Our previous work demonstrated that the microbial communities inhabiting the chimneys are fueled by carbon venting from Earth’s mantle (4). The present study identifies the genomic potential of the *Chloroflexi* and *Sulfurovum* populations to utilize formate, which may be required to make mantle-derived carbon available to the rest of the chimney ecosystem.

The Lost City biofilms that inhabit the anoxic interiors of the chimneys have been described as containing a single species, the Lost City *Methanosarcinales* phylotype (4–8, 12). The single *Methanosarcinales* MAG reported here represents 4.41% of the chimney metagenome, >5 times more abundant than the other MAGs reported here. The *Methanosarcinales* phylotype has previously been shown to dominate the anoxic, interior zones of Lost City chimneys (5, 6, 8), yet it appears to be unable to use one of the most abundant carbon sources, formate. The Lost City *Chloroflexi* MAG, in contrast, contains the genes required for using formate and may be able to provide biologically available carbon to *Methanosarcinales* and other members of the biofilm community. This is an apparent conundrum, as the *Chloroflexi* MAG is seven times less abundant than the *Methanosarcinales* MAG in the chimney sample described here. One potential explanation is that *Chloroflexi* species are highly active and able to rapidly cycle carbon while maintaining a low abundance in the biofilm community. Alternatively, the *Chloroflexi* population may be more abundant in subsurface habitats underlying the chimneys, where formate is expected to be generated (4). Future research should test these hypotheses by experimentally investigating how microbial activity in subsurface environments can influence the food and energy available to the biofilm communities of the chimneys.

## MATERIALS AND METHODS

### Sample collection.

Sample H08_080105_Bio5slurpB1 from Marker 5 was collected in 2005 during a National Oceanic and Atmospheric Administration (NOAA) Ocean Explorer cruise with the ROV *Hercules* aboard the R/V *Ronald H. Brown*. The sample was immediately placed in a sterile Whirl-Pak sample bag upon arrival on deck and stored at −80°C until analysis. DNA was extracted from the samples according to a previously published protocol (6). A previous metagenomic analysis of this sample has been published (4), but the results presented here are from a different DNA extraction and a much deeper metagenomic sequencing effort. All laboratory and bioinformatic protocols are available at https://doi.org/10.5281/zenodo.3629892. Environmental DNA was extracted with a protocol modified from that described previously (5, 7, 54). The chimney sample was thawed and homogenized with a sterile mortar and pestle at room temperature, and 0.25-g subsamples were placed in a DNA extraction buffer containing 0.1 M Tris, 0.1 M Na-EDTA, 0.1 M KH_2_PO_4_, 1.5 M NaCl, 0.8 M guanidium HCl, and 0.5% Triton X-100. For lysis, samples were subjected to one freeze-thaw cycle, incubation at 65°C for 30 min, and beating with 0.1-mm glass beads in a Mini-BeadBeater 16 (Biospec Products). Purification was performed via extraction with phenol-chloroform-isoamyl alcohol, precipitation in 3 M sodium acetate and ethanol, washing in Amicon 30K Ultra centrifugal filters, and a final cleanup with 2× SPRI beads (55). The DNA concentration after purification was approximately 10 ng/μl, as measured with a Qubit fluorometer (Thermo Fisher), with an *A*_260_/*A*_230_ ratio of 1.6 and an *A*_260_/*A*_280_ ratio of 1.8, as measured with a NanoDrop spectrophotometer (Thermo Fisher).

### Metagenome sequencing.

A Qsonica Q800R sonicator was used to fragment the metagenomic DNA to ∼500 to 700 bp. A metagenome library was constructed with 500 ng of fragmented DNA using a NEBNext Ultra DNA library preparation kit for Illumina according to the manufacturer’s instructions. Quality control and sequencing of the metagenomic libraries were conducted at the University of Utah High-Throughput Genomics Core Facility. Libraries were evaluated for quality on a Bioanalyzer DNA 1000 chip (Agilent Technologies), and then paired-end sequencing (2 × 125 bp) was performed on an Illumina HiSeq2500 platform with HiSeq (v4) chemistry. The library was multiplexed with one other library (from a second Lost City chimney sample, results from which are not reported here) on one Illumina lane, yielding 180 million read pairs (45 billion bases). Demultiplexing and conversion of the raw sequencing base-call data were performed through the CASAVA (v1.8) pipeline.

### Metagenomic analysis.

Raw sequence data were processed by the W. J. Brazelton lab to trim adapter sequences with BBDuk (part of the BBTools suite, v35.85 [56]), to remove artificial replicates, and to trim the reads based on quality. Removal of replicates and quality trimming were performed with our seq-qc package (https://github.com/Brazelton-Lab/seq-qc). Paired-end reads were assembled with the MegaHit (v1.1.1) program, using kmers of 27 to 141. The Prodigal (v2.6.3) program was run in the anonymous gene prediction mode to identify open reading frames. Functional annotation was performed using the blastp function of the Diamond (v0.9.14) program with both the prokaryotes and T10000 (addendum annotations) databases from KEGG (release 83.2) with an E value of 1e−6. Annotations were selected by the highest-quality alignment, as determined by the bit score. Binning was performed with the ABAWACA (v1.00) program (https://github.com/CK7/abawaca). Contig taxonomy was assigned with PhyloPythiaS+ (v1.4) software (20). Curation of bins was performed in the anvi’o (v4) platform, using the default visualization of bins informed by tetranucleotide frequency as well as manual inspection of the PhyloPythiaS+ taxonomic assignment. The CheckM (v1.0.5) program was used to assess bin quality (57). Completion of the KEGG modules and pathways was determined using the KEGG Mapper online tool (https://www.genome.jp/kegg/mapper.html, accessed June 2019). Coverage was determined through read mapping with the bowtie2 (v2.3.2) and bedtools (v2.25.0) genomecov programs (58, 59). Reference proteins for phylogenetic trees were downloaded from NCBI GenBank in September 2019, and descriptions of these proteins can be found in Table S5 in the supplemental material. The multiple-sequence alignments were built with the MUSCLE (v3.8.31) program (60), and the phylogenies were inferred with the RaxML (v8.2.0) program and the -f a option with 100 bootstrap replicates.

### Data availability.

All unassembled sequences related to this study are available at the NCBI Sequence Read Archive (BioSample accession number SAMN13035994), and MAG assemblies have been submitted to NCBI GenBank (BioSample accession numbers SAMN13172856 to SAMN13172858). All NCBI data may be found under BioProject accession number PRJNA577730. All SRA metadata, supplementary materials, and protocols are archived at https://doi.org/10.5281/zenodo.3629892. All custom software and scripts are available at https://github.com/Brazelton-Lab.

## Supplementary Material

Supplemental file 1

Supplemental file 2
