# Supplementary material for: Genomic Evidence for Formate Metabolism by Chloroflexi as the Key to Unlocking Deep Carbon in Lost City Microbial Ecosystems
Source: Appl Environ Microbiol. 2020 Apr 1;86(8):e02583-19. doi: 10.1128/AEM.02583-19 (PMC7117926; doi:10.1128/AEM.02583-19)
Supplement: Supplemental file 1 [file AEM.02583-19-s0001.pdf]

Supplemental Material for *Genomic Evidence for Formate Metabolism by Chloroflexi as the Key to Unlocking Deep Carbon in Lost City Microbial Ecosystems*

Julia M. McGonigle, Susan Q. Lang, William J. Brazelton

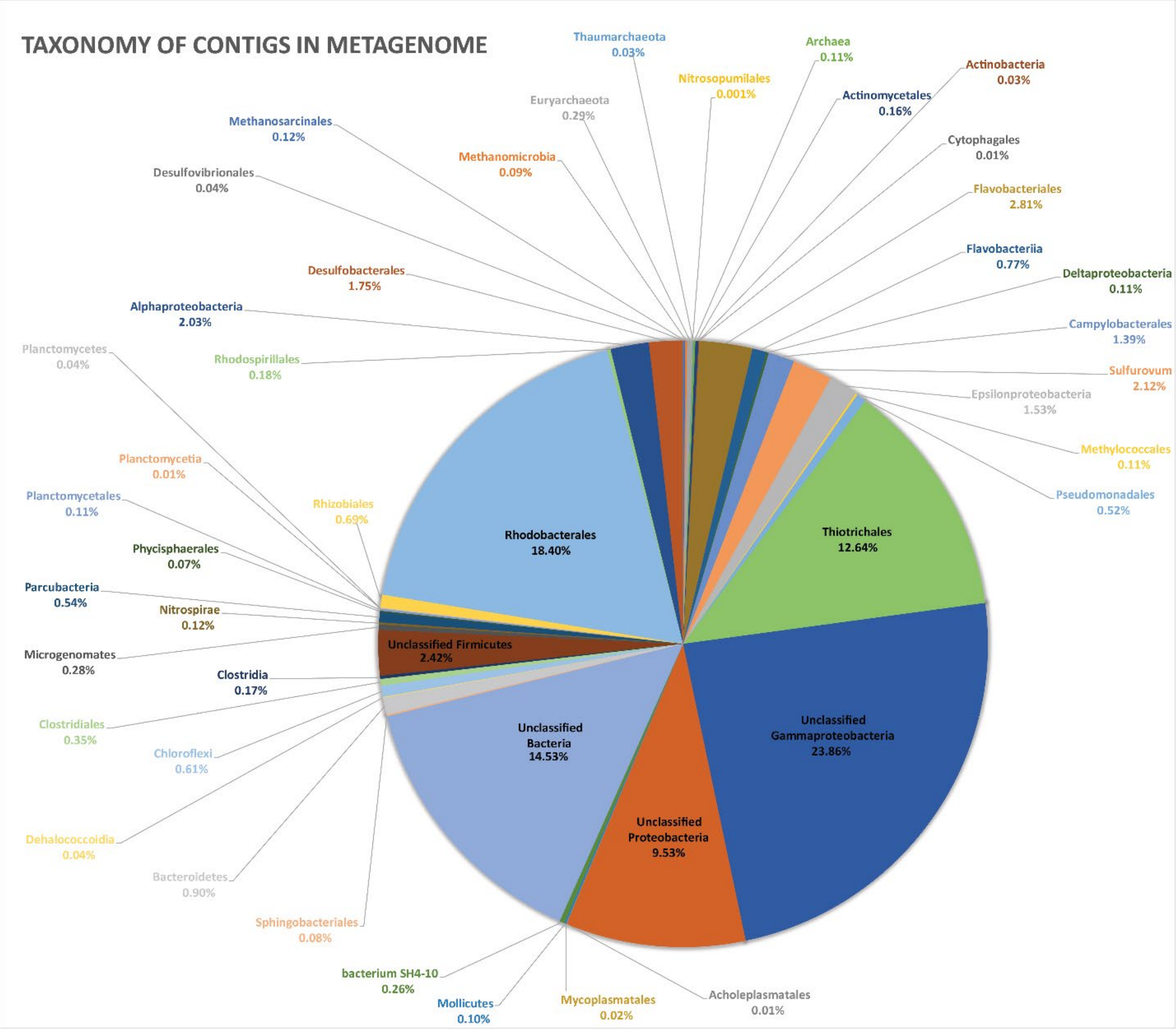

Figure S1: Coverage of assembled contigs from Lost City chimney sample near Marker 5, grouped by taxonomic assignments. PhyloPhythiaS+ taxonomic consensus for each contig is reported at the level of order. Exceptions are *Sulfurovum* and contigs with unassigned order, where the lowest possible assignment is reported.

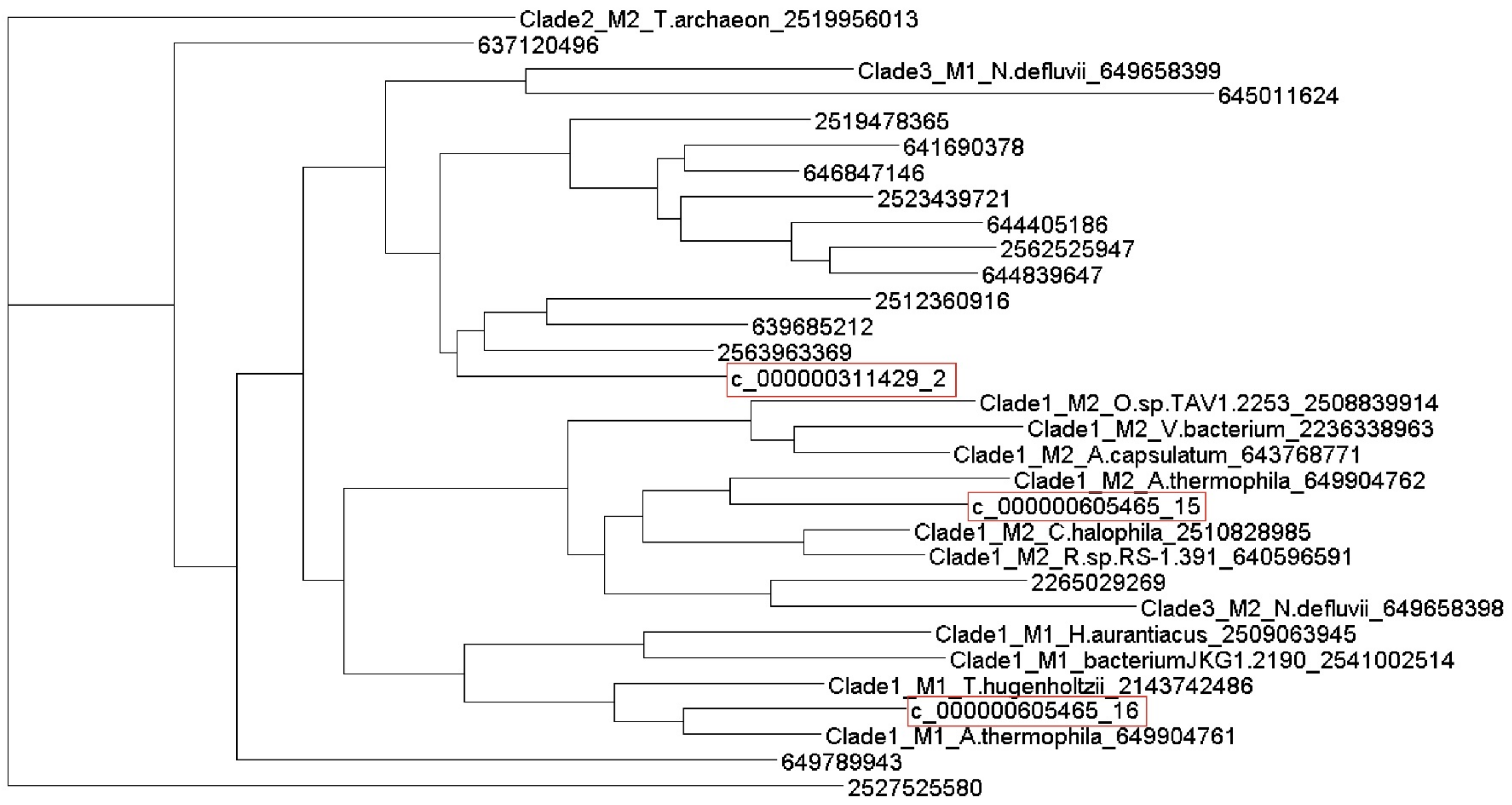

Figure S2: Phylogeny of the three *nuoM* sequences (boxed in red) found in the *Chloroflexi* MAG. The tree was built using RaxML from a subset of reference proteins published in Chadwick *et al.* (1) obtained from JGI's IMG database, accessed January 2020. Clade distinctions (Clade1_M1, Clade1_M2, Clade2_M2, Clade3_M1, Clade3_M2) are identical to those identified by Chadwick *et al.* ID numbers are the IMG Gene ID.

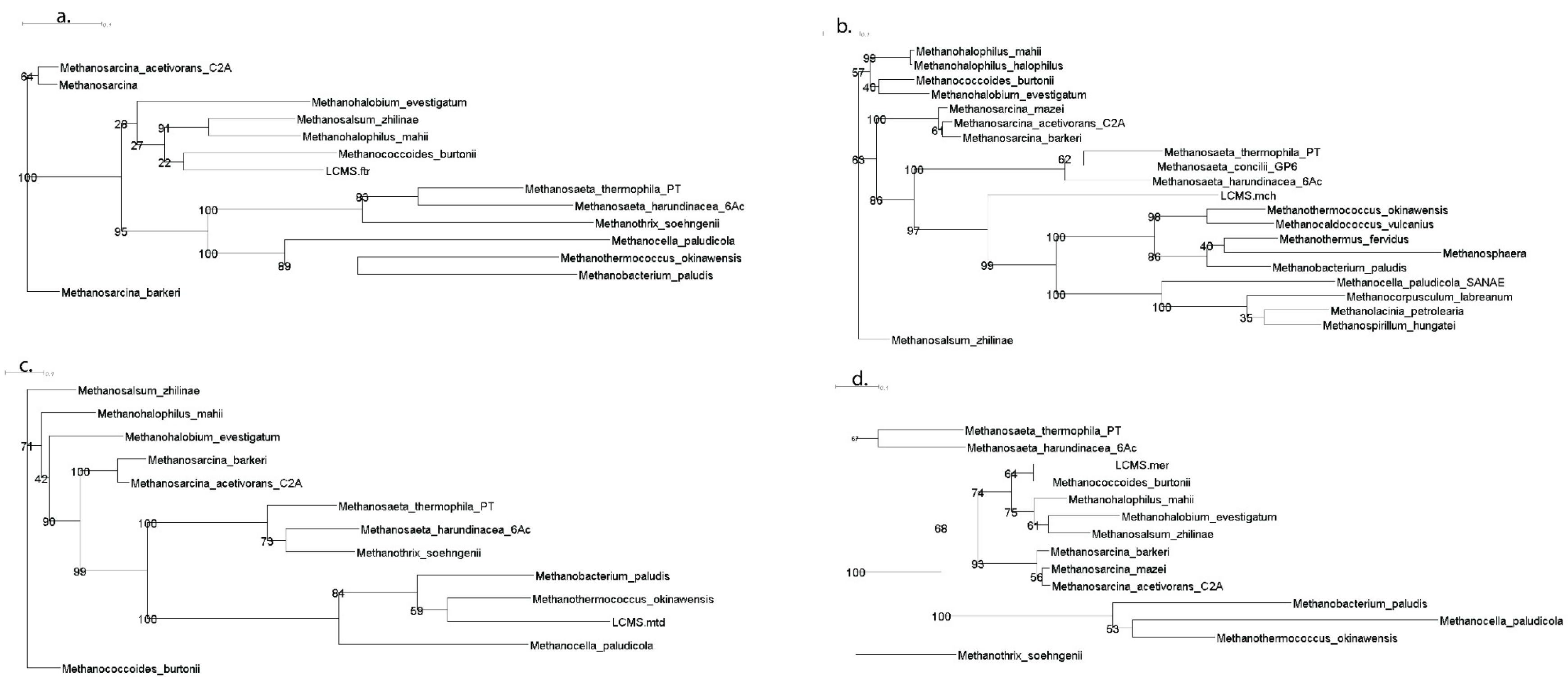

Figure S3: Phylogenetic trees of Lost City *Methanosarcinales* (LCMS) MAG genes in methanogenic pathways. **a.** formylmethanofuran-tetrahydromethanopterin formyltransferase (*ftr*), **b.** N^5N^{10}-methenyl-tetrahydromethanopterin cyclohydrolase (*mch*), **c.** F_{420}-dependent N^5N^{10}-methylene-tetrahydromethanopterin dehydrogenase (*mtd*), **d.** and F_{420}-dependent N^5N^{10}-methylene-tetrahydromethanopterin reductase (*mer*). Accession numbers for reference proteins can be found in Table S5.

Bin Id	CheckM Marker Lineage	PhyloPhythiaS+ Taxonomic Consensus	BLAST for Ribosomal S15 Protein	% ID	% Completeness	% Contamination	% of Assembly Coverage
20	k__Bacteria (UID2565)	*Planctomycetes; Phycisphaera mikurensis*	*Phycisphaerae bacterium* (MAO22150.1)	67.42%	84.09	1.14	0.37
26	k__Bacteria (UID203)	*Epsilonproteobacteria; Sulfurovum/Sulfurospirillum*	NA	NA	27.93	1.72	0.14
28	k__Bacteria (UID203)	*Proteobacteria*	*Candidatus Gracilibacteria bacterium* (QFR39383.1)	76.40%	54.31	0.86	0.10
33	k__Bacteria (UID203)	*Proteobacteria*	*candidate division WOR-3 bacterium* (OYD15091.1)	68.97%	91.38	6.11	0.84
30	k__Bacteria (UID203)	No consensus	*Candidatus Acetothermia bacterium* (HCP32314.1)	80%	63.71	9.48	0.62
32	k__Bacteria (UID203)	No consensus	*Nitrospiraceae bacterium* (HCZ11968.1)	71.55%	18.79	0	0.05
36	k__Bacteria (UID203)	*Gammaproteobacteria*	*Candidatus Gracilibacteria bacterium* (QFR39383.1)	77.53%	46.11	1.57	0.06

Table S1: Taxonomic assignment of MAGs greater than 18% complete and under 10% contamination as determined by CheckM for Lost City chimney sample from Marker 5. PhyloPhythiaS+ taxonomic consensus for contigs is reported at the deepest observed level if one third or more of the contigs in the MAG were assigned the same taxonomic grouping. The BLAST search was done using the NCBI nr database; hit with highest percent identities is reported.

	Chloroflexi MAG	Sulfurovum MAG	LCMS MAG
**% Completion**	70	95.9	84.87
**% Contamination**	4.73	2.19	5.26
**# contigs (>= 0 bp)**	300	82	385
**# contigs (>= 1000 bp)**	9	82	379
**# contigs (>= 5000 bp)**	265	72	191
**# contigs (>= 10000 bp)**	172	62	43
**# contigs (>= 25000 bp)**	48	30	4
**# contigs (>= 50000 bp)**	2	9	0
**Total length (>= 0 bp)**	4327445	1944029	2369335
**Total length (>= 1000 bp)**	4327445	1944029	2366329
**Total length (>= 5000 bp)**	4178990	1908505	1661266
**Total length (>= 10000 bp)**	3489585	1835568	645388
**Total length (>= 25000 bp)**	1558923	1334736	106887
**Total length (>= 50000 bp)**	133530	563213	0
**Largest contig**	67383	108728	29121
**N50**	18477	37169	6717
**total protein-encoding genes**	3936	2036	2324
**# KEGG annotated genes**	3316	1838	1796
**# genes no annotation**	620	198	528
**# complete KEGG modules**	33	53	33
**# incomplete KEGG modules**	39	24	28
**% of Assembly Coverage**	0.62	0.41	4.41

Table S2: Assembly quality statistics for each MAG.

nifH Clone ID	contigID	% ID	Alignment Length (bp)
1537.15	c_000000344721	97.15	386
L74	c_000000344721	95.87	387
1537.102	c_000000344721	98.19	387
1537.52	c_000000344721	95.87	387
L7	c_000000344721	96.38	387

Table S3: BLAST results for previously sequenced *nifH* genes.

mcrA Clone ID	contigID	% ID	Alignment Length (bp)
LCM1443-49	c_000000702168	98.90	726
LCM1228-3	c_000000702168	99.18	728
LCM1404-22	c_000000702168	96.80	719

Table S4: BLAST results for previously sequenced *mcrA* genes.

Enzyme	Accession number	Organism
Ftr	Mhar_2214	*Methanosaeta harundinacea* 6Ac
Ftr	YP_004383947	*Methanosaeta concilii* GP6
Ftr	YP_844050	*Methanosaeta thermophila* PT
Ftr	NP_614984	*Methanosarcina acetivorans* C2A
Ftr	YP_304532	*Methanosarcina barkeri* Fusaro
Ftr	NP_633345	*Methanosarcina mazei* Go1
Ftr	YP_566370	*Methanococcoides burtonii* DSM 6242
Ftr	YP_003727460	*Methanohalobium evestigatum* Z-7303
Ftr	YP_003543165	*Methanohalophilus mahii* DSM 5219
Ftr	YP_004617074	*Methanosalsum zhilinae* DSM 4017
Ftr	YP_004521004	*Methanobacterium sp.* SWAN-1
Ftr	YP_003356088	*Methanocella paludicola* SANAE
Ftr	YP_004575931	*Methanothermococcus okinawensis* IH1
Mch	Mhar_2174	*Methanosaeta harundinacea* 6Ac
Mch	YP_004383804	*Methanosaeta concilii* GP6
Mch	YP_842689	*Methanosaeta thermophila* PT
Mch	NP_616637	*Methanosarcina acetivorans* C2A
Mch	YP_305738	*Methanosarcina barkeri* Fusaro
Mch	NP_634677	*Methanosarcina mazei* Go1
Mch	YP_565374	*Methanococcoides burtonii* DSM 6242
Mch	YP_003727251	*Methanohalobium evestigatum* Z-7303
Mch	YP_003542289	*Methanohalophilus mahii* DSM 5219
Mch	YP_004616412	*Methanosalsum zhilinae* DSM 4017
Mch	YP_004519292	*Methanobacterium sp.* SWAN-1
Mch	YP_003247696	*Methanocaldococcus vulcanius* M7
Mch	YP_003356187	*Methanocella paludicola* SANAE
Mch	YP_001029842	*Methanocorpusculum labreanum* Z
Mch	YP_003893284	*Methanoplanus petrolearius* DSM 11571
Mch	YP_448258	*Methanosphaera stadtmanae* DSM 3091
Mch	YP_501925	*Methanospirillum hungatei* JF-1
Mch	YP_004577331	*Methanothermococcus okinawensis* IH1
Mch	YP_004003819	*Methanothermus fervidus* DSM 2088
Mer	Mhar_0856	*Methanosaeta harundinacea* 6Ac
Mer	YP_004384673	*Methanosaeta concilii* GP6
Mer	YP_842643	*Methanosaeta thermophila* PT
Mer	NP_618605	*Methanosarcina acetivorans* C2A
Mer	YP_303818	*Methanosarcina barkeri* Fusaro

Mer	NP_632652	*Methanosarcina mazei* Go1
Mer	YP_566974	*Methanococcoides burtonii* DSM 6242
Mer	YP_003727762	*Methanohalobium evestigatum* Z-7303
Mer	YP_003542654	*Methanohalophilus mahii* DSM 5219
Mer	YP_004615267	*Methanosalsum zhilinae* DSM 4017
Mer	YP_004520699	*Methanobacterium sp.* SWAN-1
Mer	YP_003357736	*Methanocella paludicola* SANAE
Mer	YP_004575971	*Methanothermococcus okinawensis* IH1
Mtd	Mhar_1470	*Methanosaeta harundinacea* 6Ac
Mtd	YP_004383615	*Methanosaeta concilii* GP6
Mtd	YP_843238	*Methanosaeta thermophila* PT
Mtd	NP_619291	*Methanosarcina acetivorans* C2A
Mtd	YP_304643	*Methanosarcina barkeri* Fusaro
Mtd	NP_619291	*Methanosarcina mazei* Go1
Mtd	YP_565622	*Methanococcoides burtonii* DSM 6242
Mtd	YP_003727361	*Methanohalobium evestigatum* Z-7303
Mtd	YP_003541849	*Methanohalophilus mahii* DSM 5219
Mtd	YP_004615335	*Methanosalsum zhilinae* DSM 4017
Mtd	YP_004518857	*Methanobacterium sp.* SWAN-1
Mtd	YP_003356368	*Methanocella paludicola* SANAE
Mtd	YP_004576424	*Methanothermococcus okinawensis* IH1

Table S5: Accession numbers for reference proteins used in Figure S2.

Dataset S1: DatasetS1.xlsx - Complete and incomplete KEGG modules for the *Methanosarcinales*, *Sulfurovum*, *Chloroflexi* MAGs.
